# Multi‐fidelity Monte Carlo fusion for BNCT dose calculation using a dual‐branch network

**DOI:** 10.1002/mp.70646

**Published:** 2026-08-24

**Authors:** Leiming Li, Xin Hong, Yongquan Wang, Gan Huang, Zhiqiang Wu, Yingjie Xiao, Hiroki Tanaka, Hiroshi Watabe

**Affiliations:** ^1^ Graduate School of Biomedical Engineering Tohoku University Sendai Japan; ^2^ Research Center for Accelerator and Radioisotope Science Tohoku University Sendai Japan; ^3^ School of Nuclear Science and Technology Lanzhou University Lanzhou China; ^4^ Division of Particle Radiation Medical Physics Institute for Integrated Radiation and Nuclear Science Kyoto University Osaka Japan

**Keywords:** boron neutron capture therapy, deep learning, dose calculation, Monte Carlo simulation

## Abstract

**Background:**

Boron neutron capture therapy (BNCT) is a binary radiotherapy that selectively kills tumor cells. Its clinical implementation relies on Monte Carlo (MC)‐based treatment planning systems, which are time‐consuming and limit clinical workflow efficiency.

**Purpose:**

To address this limitation, we propose a Multi‐Fidelity MC‐based Dual‐Branch Network (MFMC‐DBN) that leverages complementary information from low‐ and high‐fidelity MC simulations through dual‐branch feature fusion, enabling accurate and efficient BNCT dose calculation.

**Methods:**

The model was trained, validated, and tested using clinical data from 112 glioblastoma patients. Low‐statistics coarse and fine mesh MC dose distributions (6.5×10^6^ particle histories) and patient CT information were used to predict three‐dimensional dose distributions. Performance was compared with an MC denoising method and a conventional neural network (NN) approach.

**Results:**

MFMC‐DBN outperformed both the NN and MC denoising methods across evaluated metrics. It achieved a mean absolute percentage error (MAPE) of 1.98% in the gross tumor volume (GTV). Organ‐specific MAPEs ranged from 1.1% to 3.9%, with a maximum mean absolute error (MAE) of0.28 Gy (RBE). Gamma analysis further showed high spatial agreement, with passing rates of 99.8% within the patient contour and 99.3% in the GTV under the 3%/3 mm criterion. The proposed method maintained high dosimetric accuracy while substantially accelerating BNCT dose calculation.

**Conclusions:**

MFMC‐DBN effectively integrates multi‐fidelity MC information through a dual‐branch attention mechanism, enabling rapid and accurate 3D BNCT dose prediction. By significantly reducing computation time compared with full MC simulations while preserving dosimetric fidelity, these results support the feasibility of the proposed framework for accelerated BNCT dose calculation under a fixed beam configuration.

## 1 INTRODUCTION

Boron neutron capture therapy (BNCT) is a type of binary radiotherapy based on the ^10^B (n, α) ^7^Li reaction, which releases high‐linear energy transfer (LET) α particles and ^7^Li ions.^1^ The therapeutic selectivity of BNCT arises from the preferential accumulation of ^10^B in tumor cells and the very short range of the reaction products, which confines radiation damage primarily to boron‐containing tissues. In recent years, BNCT has garnered increasing attention^2^ due to its high selectivity for tumor tissue, low toxicity, and significant therapeutic efficacy. Clinical studies^3^, ^4^, ^5^ have demonstrated that BNCT exhibits unique advantages compared with conventional treatments such as surgery, chemotherapy, and photon radiotherapy, especially for head and neck malignancies and glioblastoma.

The implementation of BNCT relies on the treatment planning system (TPS), which simulates the treatment process and calculates patient‐specific dose distributions for individualized planning. Therefore, accurate dose calculation is the basis for TPS. Current TPSs, such as the JAEA Computational Dosimetry System (JCDS),^6^ the Simulation Environment for Radiotherapy Applications (SERA),^7^ NCTPlan,^8^ and NeuCure® Dose Engine,^9^ which integrates the Particle and Heavy Ion Transport Code System (PHITS),^10^ utilize Monte Carlo (MC) methods for their high accuracy. However, obtaining reliable results from MC simulations is extremely time‐consuming,^11^ thereby substantially limiting the clinical efficiency of BNCT.

In order to reduce the time required for BNCT dose calculations, developing alternative algorithms to replace conventional MC methods has become an important research direction. Recently, neural networks (NN) have been widely applied in the medical domains, including automated radiotherapy planning and dose prediction. In photon radiotherapy, studies have shown that using NN to predict three‐dimensional (3D) dose distributions is feasible.^12^ In BNCT, one study^13^ reported that using NN to predict dose distributions reduced the computation time required by conventional MC methods from approximately 6 h to less than 1 second, thereby significantly improving efficiency. Another study^14^ trained an NN using coarse mesh (32 × 32 × 32) MC results as inputs and adjusted the loss function to predict fine mesh (128 × 128 × 128) MC dose distributions. This strategy reduced the mean absolute percentage error (MAPE) in the gross tumor volume (GTV) from 9.5% to 3.3%, compared with an NN trained without MC inputs. These findings suggest that incorporating MC results as inputs can enhance both the accuracy and interpretability of NN predictions. However, the use of low‐statistics MC results for model development and training in 3D BNCT dose prediction has not been thoroughly investigated. Further methodological development is still needed to improve both prediction accuracy and interpretability.

We propose a Multi‐Fidelity MC–based Dual‐Branch Network (MFMC‐DBN) algorithm for BNCT dose calculation, enabling rapid and accurate prediction of 3D dose distributions by leveraging low‐statistics multi‐fidelity MC results. This algorithm integrates the global consistency of coarse mesh MC results with high precision local details in high‐dose regions of fine mesh MC results. Through dual‐branch feature fusion and attention mechanisms, the algorithm effectively combines the advantages of both MC fidelities while maintaining physical fidelity, providing a novel and efficient strategy for accurate dose prediction in BNCT TPS. Furthermore, this approach is highly compatible with hardware acceleration (e.g., GPU parallelization); by refining the low‐statistics outputs from fast hardware simulators, it provides a synergistic foundation for real‐time BNCT treatment planning. Nevertheless, this study was conducted under a fixed irradiation geometry, and further validation under varying beam angles and source‐to‐skin distances is required before broader TPS implementation.

## 2 MATERIALS AND METHODS

### 2.1 Patient data and preprocessing

In this work, radiotherapy data from 112 glioblastoma patients treated at Massachusetts General Hospital^16^, ^17^ were collected, with the dataset provided by TCIA.^15^ For each patient, treating radiation oncologists manually delineated the radiotherapy target volumes, including the GTV and clinical target volume (CTV), as well as organs at risk (OARs), including the brainstem, eyes, optic chiasm, optic nerves, cochleae, and lacrimal glands. These target volumes and OARs were located in the intracranial region, consistent with the anatomical characteristics of glioblastoma cases. The skin was defined as a 3 mm layer inside the patient contour.^9^ The dataset does not include clinical prescription dose information.

The average volumes (mean ± standard deviation) of the representative target and OAR structures in the dataset were as follows: GTV, 130.88 ± 73.40 cm^3^; brainstem, 27.40 ± 5.30 cm^3^; eyes, 17.16 ± 1.61 cm^3^; optic nerves, 1.31 ± 0.30 cm^3^; optic chiasm, 0.50 ± 0.23 cm^3^; cochleae, 0.18 ± 0.03 cm^3^; and lacrimal glands, 0.96 ± 0.37 cm^3^. For volume calculation, overlapping regions between the OARs and target volumes were excluded to avoid ambiguity in anatomical assignment.

During preprocessing, voxels outside the patient contour were labeled as air, and all CT images were resampled to a uniform resolution of 128 × 128 × 128 voxels, with a voxel size of 2.6 × 2.6 × 2.5 mm^3^. The dataset was randomly split into 90 training, 9 validation, and 13 test cases to ensure an unbiased evaluation.

### 2.2 Monte Carlo dose calculations

The PHITS 3.31 MC code was used for dose calculation. To construct the voxel model, the Hounsfield unit (HU) value of each voxel was converted into material density and elemental composition.^18^ Based on the organ masks, the ^10^B distribution was assigned according to tissue type, with concentrations of 60 ppm in tumors, 18 ppm in normal tissues, 25 ppm in the skin, and 0 ppm in bone. The voxel model had the same spatial resolution as the CT data (128 × 128 × 128, voxel size 2.6 × 2.6 × 2.5 mm^3^).

**FIGURE 1:**
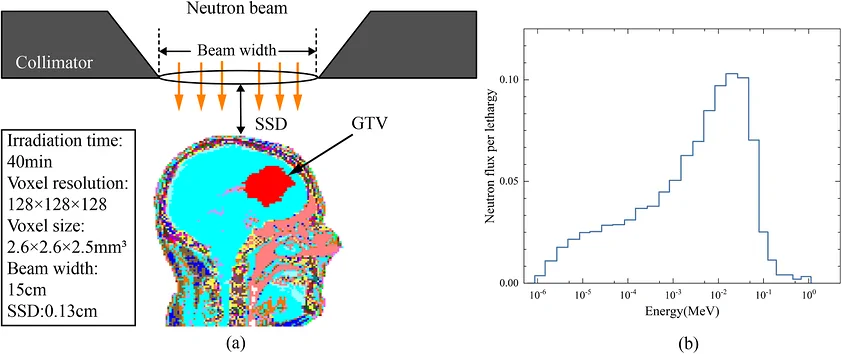
PHITS simulation configuration used for BNCT dose calculation. (a) Schematic illustration of the irradiation setup. (b) Energy spectrum of the incident epithermal neutron beam used in the simulations.

The neutron source was the Kyoto University Cyclotron‐Based Epithermal Neutron Source (C‐BENS),^19^ operated at 1 mA to provide an epithermal neutron beam. The source characteristics, including the neutron energy spectrum and beam geometry, were implemented based on previously reported C‐BENS data.^19^ The corresponding energy spectrum, beam shape, beam size, irradiation field, and irradiation geometry are shown in Figure 1.

BNCT dose calculations consider four absorbed dose components:^20^ boron dose (D_B_), nitrogen dose (D_N_), hydrogen dose (D_H_), and gamma dose (D_γ_). In this study, each component was calculated by multiplying the neutron flux tallies with the corresponding kerma factors. Due to the different biological effects of the four absorbed dose components, the relative biological effectiveness (RBE) or compound biological effectiveness (CBE) factors were applied (Table 1).^9^

**TABLE 1:** Relative biological effectiveness and compound biological effectiveness factors used in this study for calculating the RBE‐weighted dose.

Tissue type	CBE	RBE_N_	RBE_H_	RBE_γ_
Tumor	3.8	2.9	2.4	1
Skin	2.5	2.9	2.4	1
Normal tissues	1.34	2.9	2.4	1

The RBE‐weighted dose (D_RBE)^21^ represents the equivalent biological effects and was calculated by multiplying each dose component with its corresponding RBE or CBE factor and then summing the results (Eq.1).

(1) $$\begin{array}{ccc} D_{RBE} & = & CBE_{B}\times D_{B}+RBE_{H}\times D_{H}\\ & & +\;RBE_{N}\times D_{N}+RBE_{\gamma }\times D_{\gamma } \end{array}$$

In MC simulations, a larger number of particles in each tally mesh facilitates statistical convergence. Based on this, the voxel model was divided into two different tally meshes: a fine mesh (128 × 128 × 128, voxel size 2.6 × 2.6 × 2.5 mm^3^) and a coarse mesh (32 × 32 × 32, voxel size 10.4 × 10.4 × 10 mm^3^). When selecting the voxel size for the fine mesh, we referred to the spatial resolutions adopted in previous related studies^9^, ^13^, ^14^ and also considered the computational resources required for performing Monte Carlo dose calculations across the entire dataset. Based on these considerations, a voxel size of 2.6 × 2.6 × 2.5 mm^3^ was ultimately selected for the fine mesh. It should be noted that, for some clinical cases, this resolution may still be relatively coarse and could introduce volume‐averaging effects, thereby affecting the accuracy of local dose distributions. To address this issue, the potential impact of volume averaging under a finer voxel grid is further analyzed in the Discussion section.

The coarse‐mesh voxel size was introduced as a lower‐fidelity representation to improve statistical stability and accelerate dose prediction under low‐statistics conditions. Accordingly, the selection of the coarse‐mesh resolution was required to satisfy several criteria: (1) the resulting dose distributions should exhibit sufficiently low statistical uncertainty; (2) the coarse mesh should provide a clear computational efficiency advantage over the fine mesh; and (3) to enable structured correspondence and facilitate feature fusion with the fine‐mesh input, the coarse‐mesh grid size should be an integer divisor of the fine‐mesh resolution, such as 64^3^, 32^3^ or 16^3^.

Based on these considerations, different coarse‐mesh configurations were evaluated in terms of their trade‐offs. A 64^3^ grid would offer only limited computational efficiency gains compared with the fine mesh, whereas a 16^3^ grid would result in excessively large voxel sizes and, consequently, a more pronounced loss of spatial resolution. Considering statistical uncertainty, spatial resolution, and computational efficiency, a 32^3^ grid was selected as a balanced choice, corresponding to a voxel size of 10.4 × 10.4 × 10 mm^3^. In the present framework, the coarse‐mesh result is not used as the final dose output but rather serves as an auxiliary source of global contextual information for the network. This design reflects a practical compromise among spatial resolution, MC statistical uncertainty, and computational cost within the present proof‐of‐concept framework.

Under low‐statistics conditions, simulations were performed on both the coarse and fine meshes. The coarse mesh results provide more stable dose distributions with good global consistency but lack local detail; whereas the fine mesh results capture high‐precision local details in high‐dose regions but are less accurate in low‐dose regions due to insufficient particle counts. As the reference “gold‐standard” results, simulations were performed on the fine mesh using a sufficiently large number of particle histories to obtain reliable outcomes. The number of particle histories for the low‐statistics simulations was 6.5 × 10^6^, whereas that for the gold‐standard simulations was 1 × 10^8^. The 1 × 10^8^‐particle simulation was used as the reference dataset in this study. The simulations employed the JENDL‐4 nuclear data library, accounting for photon and neutron interactions relevant to BNCT. 3D dose distributions were first obtained under reference conditions (1 ppm ^10^B, 1% H, and 1% N) and were then scaled according to voxel‐specific ^10^B concentrations and hydrogen/nitrogen mass fractions to obtain the actual dose distributions.

### 2.3 MFMC‐DBN algorithm architecture

**FIGURE 2:**
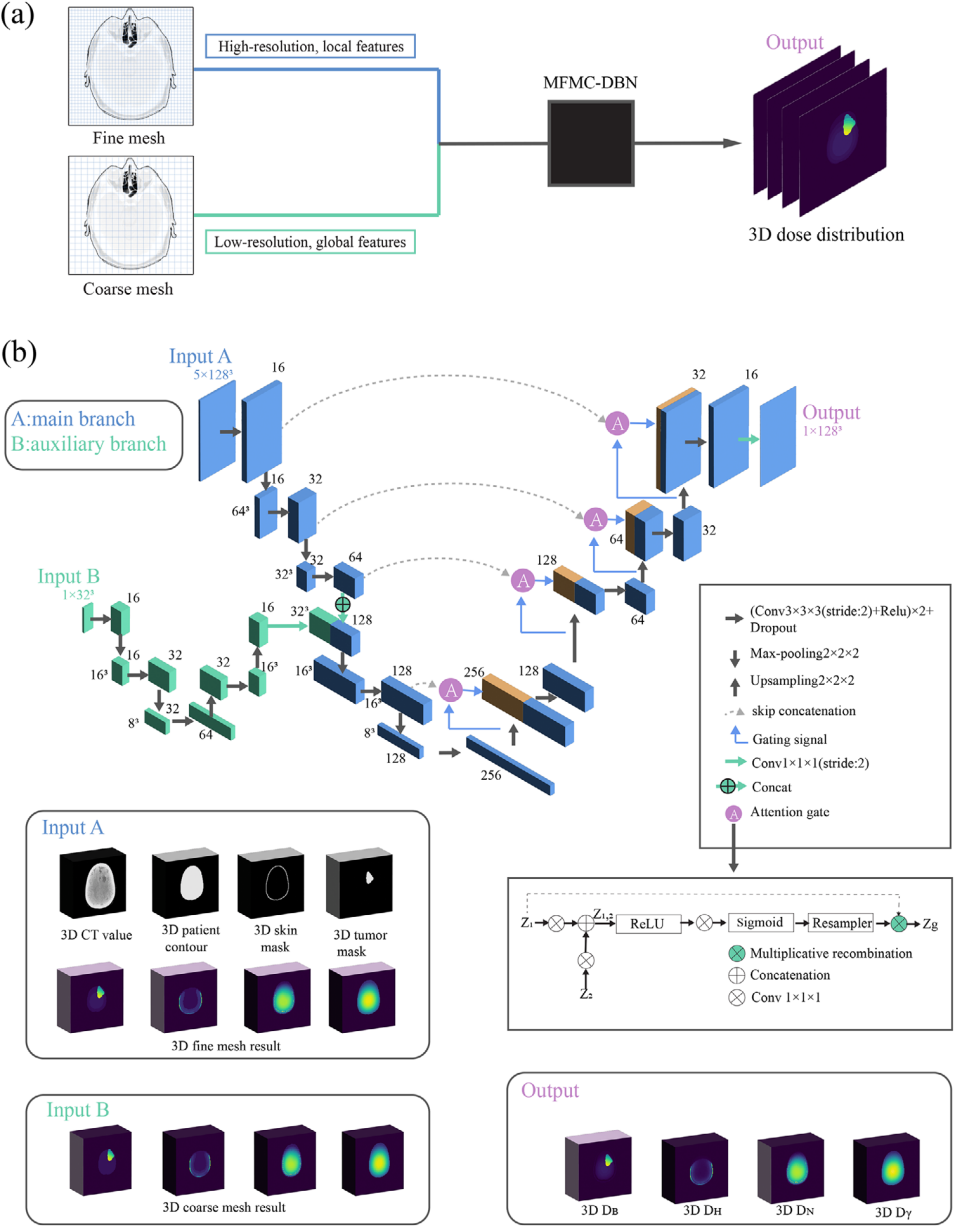
Overview of the proposed MFMC‐DBN for BNCT dose prediction. (a) Conceptual rationale for using multi‐fidelity MC inputs, where the fine mesh provides local high‐resolution information and the coarse mesh provides global contextual information. (b) Detailed architecture of the dual‐branch neural network.

MFMC‐DBN is a deep learning framework for rapid 3D BNCT dose prediction that integrates low‐statistics MC results from both fine and coarse tally meshes. As illustrated in Figure 2a, the fine mesh provides high‐resolution local dose information, whereas the coarse mesh provides more stable global dose patterns under low‐statistics conditions. As shown in Figure 2b, MFMC‐DBN consists of a main branch and an auxiliary branch. The fine‐mesh tally is used as the input to the main branch to preserve local detail and improve prediction accuracy in high‐dose regions, while the coarse‐mesh tally is fed into the auxiliary branch to provide global contextual information and reduce prediction errors in low‐dose regions. Feature fusion is performed at the third level of the main encoder.

In terms of network architecture, the main input is a five‐channel tensor of size 128^3^, whereas the auxiliary input is a single‐channel tensor of size 32^3^. The main branch employs a five‐level encoder. Each level consists of two 3 × 3 × 3 convolutional layers followed by ReLU activation, with dropout applied after the second convolution and increasing progressively from 0.10 to 0.30. Spatial downsampling is performed using 2 × 2 × 2 max‐pooling with a stride of 2, reducing the feature‐map size from 128^3^ to 4^3^ while increasing the number of channels from 16 to 256. The auxiliary branch extracts global contextual features through three convolutional blocks with 16, 32, and 64 channels, respectively. Each block contains two 3×3×3 convolutional layers, ReLU activation, and dropout at the end of the block. The auxiliary features are downsampled to 8^3^ by max‐pooling and then upsampled to 32^3^, producing 64‐channel feature maps. These feature maps are concatenated with the output of the third encoder level of the main branch along the channel dimension. At this fusion level, the main‐branch feature map has a dimension of 32 × 32 × 32 × 64, and the auxiliary‐branch feature map has the same spatial dimension of 32 × 32 × 32 with 64 channels. After channel‐wise concatenation, the fused feature map has a dimension of 32×32×32×128. Because the coarse mesh is an integer downsampling of the fine mesh, each coarse voxel corresponds to 4 × 4 × 4 fine‐mesh voxels. Therefore, the auxiliary feature map and the downsampled main‐branch feature map share the same physical field of view and are spatially aligned at the point of concatenation.

During decoding, the decoder progressively recovers the spatial resolution through four upsampling stages. At each stage, an attention gate is applied to the corresponding skip‐connection features to selectively emphasize relevant information. Specifically, the decoder features are first transformed by a 1 × 1 × 1 convolution to generate gating signals. The corresponding encoder features are processed by a 2 × 2 × 2 convolution to match the spatial scale of the gating signals, and the two are then added elementwise. After ReLU and sigmoid activation, a single‐channel attention map is obtained. This attention map is subsequently upsampled and replicated across channels to match the original encoder features, and then multiplied elementwise with the skip features. In this way, the network can emphasize regions that are more relevant to dose prediction while suppressing irrelevant or noisy features, thereby improving reconstruction accuracy during decoding.

The main branch of the network receives five input channels: (1) low‐statistics fine‐mesh MC results, (2) patient mask, (3) CT values, (4) skin mask, and (5) tumor mask. The auxiliary branch takes the low‐statistics coarse‐mesh MC results as input to provide global dose‐distribution priors. For loss function selection, we compared mean squared error (MSE, Equation 2), mean absolute error (MAE), log‐cosh loss, and weighted MSE (MSE_W_, Equation 3).

(2) $$Loss_{MSE}=\frac{1}{N}\sum_{i=1}^{N}{\left(D_{pred}^{i}-D_{true}^{i}\right)}^{2}$$

(3) $$\begin{array}{ccc} Loss_{MSE_{w}} & = & MSE(d_{pred},D_{true})\\ & & +w_{Skin}^{2}\times MSE\left(D_{skinpred},D_{skintrue}\right) \end{array}$$

Weighted MSE provided only limited improvement in the skin region and tended to reduce the overall stability of prediction. In contrast, standard MSE yielded more stable performance across the entire dose distribution, particularly in clinically important high‐dose regions. Therefore, standard MSE was selected as the final loss function. The proposed models were implemented in PyTorch and trained from scratch on a single NVIDIA RTX 4090 GPU for 300 epochs with a batch size of 3. The Adam optimizer was used with default parameters (β1 = 0.9, β2 = 0.999). To satisfy the practical requirements of BNCT dose calculation, four independent networks were trained to predict the boron, hydrogen, nitrogen, and photon dose components (Gy) under the reference conditions of 1 ppm ^10^B, 1% H, and 1% N. This component‐wise strategy was adopted in the present proof‐of‐concept study because the four BNCT dose components have different physical origins, spatial distributions, dose ranges, and statistical‐noise characteristics, allowing each component to be learned and evaluated independently. In a unified multi‐channel or multi‐head network, these component‐specific differences may introduce optimization imbalance among dose components if appropriate normalization or task‐weighting strategies are not used. Therefore, separate component‐wise models were used as a conservative strategy to improve training stability and facilitate component‐specific evaluation. The final RBE‐weighted dose was then computed using Equation (1). Before training, each input dose component was normalized to the range of [0, 1] using its global maximum value in the training dataset.

### 2.4 Performance evaluation

To evaluate the performance of the MFMC‐DBN algorithm, we compared it with two existing methods: (1) a NN method based on 3D U‐Net without low‐statistics MC inputs, hereafter referred to as the NN method (without attention gate mechanisms),^13^ and (2) an MC denoising method based on 3D Attention U‐Net. The MC denoising method was implemented using the same 3D Attention U‐Net architecture as the main branch of MFMC‐DBN, but without the auxiliary coarse‐mesh branch and feature‐fusion module and used the same low‐statistics fine‐mesh MC dose distributions generated with 6.5×10^6^particle histories as input. It was trained to predict the corresponding high‐statistics MC dose distributions generated with 1×10^8^particle histories by minimizing voxel‐wise MSE. Its objective was to reduce statistical fluctuations in the low‐statistics MC results while preserving the underlying spatial dose distribution. This comparison was included to evaluate whether the additional low‐statistics coarse‐mesh branch in MFMC‐DBN could provide useful complementary information beyond a denoising‐only strategy based on the same fine‐mesh MC input. The main difference between MC denoising and MFMC‐DBN is the additional coarse‐mesh branch used in MFMC‐DBN. Except for the input of low‐statistics MC results, all other input channels in these two methods were identical to those used in MFMC‐DBN. The performance was first assessed at the voxel level using dose deviation (Ddev) and relative deviation (Rdev), which are defined as:

(4) $$D_{dev}=D_{p}-D_{MC}$$

(5) $$R_{dev}=\frac{\left|D_{p}-D_{MC}\right|}{D_{MC}}$$

(6) $$HI=\frac{D_{1}-D_{99}}{D_{50}}$$

where D_p_​ represents the dose predicted by the algorithm and D_MC_​ is the ground‐truth dose from the full MC simulation. Subsequently, the overall accuracy within different organs was quantified using the standard metrics of MAE and MAPE, calculated across all voxels within each organ. The method (1) utilizes a four‐channel input comprising the CT values, patient mask, skin mask, and tumor mask. The method (2) builds upon this by additionally incorporating the low‐statistics fine mesh MC result as a fifth input channel. In addition, the dose–volume histograms (DVHs) obtained from the MFMC‐DBN algorithm were compared with those from MC simulations. For all patients, the relative deviations of the doses received by 99% (D99), 98% (D98), 50% (D50), 2% (D2), and 1% (D1) of the GTV, as well as the homogeneity index^14^ (HI, see Equation 6), were evaluated. In addition, gamma analysis was performed to evaluate the spatial agreement between the predicted and reference dose distributions. Considering the voxel size used in this study (2.6 × 2.6 × 2.5 mm^3^), a 3%/3 mm criterion was adopted. The statistical uncertainty of the ground‐truth dose is also an important factor that may affect the interpretation of prediction errors, particularly in low‐dose regions. To assess the reliability of the 1 × 10^8^‐history reference MC dose distributions, we summarized the statistical uncertainty of the RBE‐weighted dose at the organ level. For each target and OAR structure, the uncertainty was reported using the mean ± standard deviation (SD), median, and 95th percentile. The mean ± SD was used to describe the overall uncertainty level and its variability across cases, the median represented the typical uncertainty level within the structure, and the 95th percentile was used to characterize the upper range of uncertainty while reducing the influence of extreme outlier voxels.

## 3 RESULTS

**FIGURE 3:**
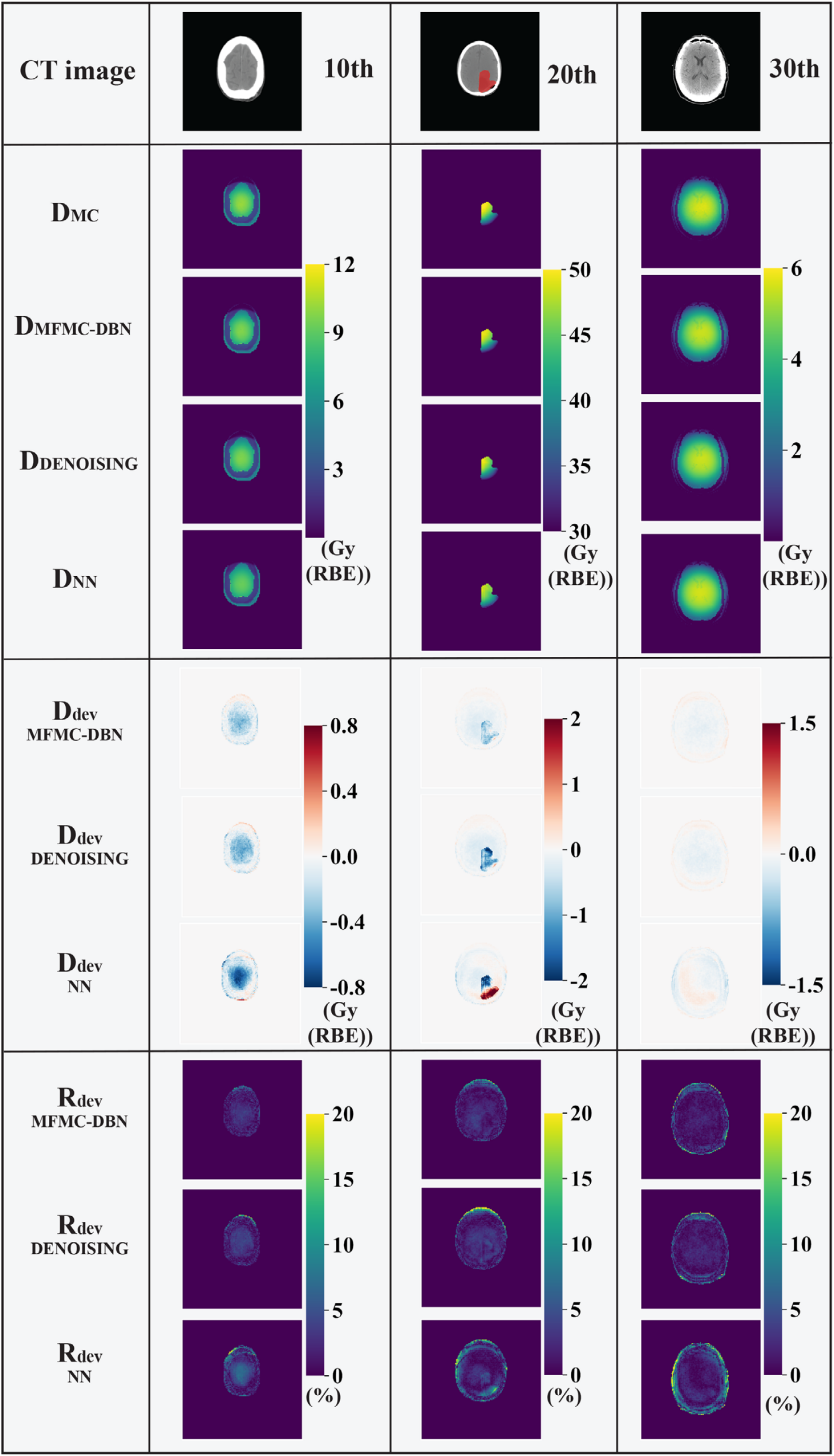
Representative comparison of CT images, dose distributions, dose deviations, and relative deviations for the 10th, 20th, and 30th slices using MFMC‐DBN, MC denoising, and NN. The red region overlaid on the CT image indicates the tumor region. From left to right, the three main columns correspond to the 10th, 20th, and 30th slices, respectively. For each slice, the CT image, MC reference dose distribution, predicted dose distributions, absolute dose deviations, and relative dose deviations are shown from top to bottom. Dose values and absolute deviations are shown in Gy (RBE), and relative deviations are shown in percentage (%).

This section presents the dose prediction results of the proposed MFMC‐DBN method. Figure 3 shows representative CT images, predicted dose distributions, dose deviations, and relative deviations for the 10th, 20th, and 30th slices obtained using MFMC‐DBN, MC denoising, and NN. Visually, MFMC‐DBN provides closer agreement with the reference dose distribution than the other two methods, with improved performance in both high‐dose and low‐dose regions.

**FIGURE 4:**
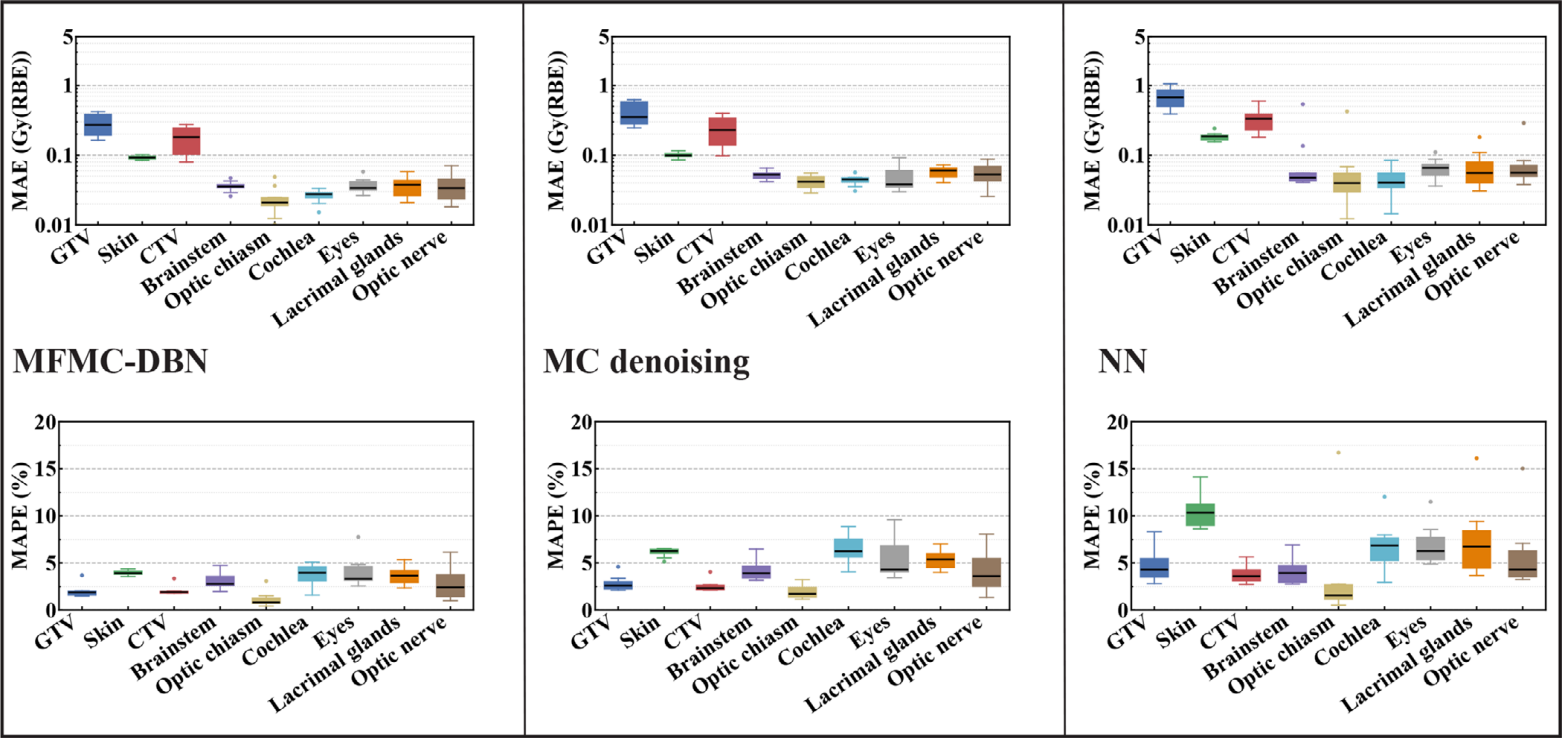
Organ‐wise comparison of mean absolute error and mean absolute percentage error for MFMC‐DBN, MC denoising, and NN. Error bars indicate 95% confidence intervals.

The estimated MC statistical uncertainty of the 1×10^8^‐history RBE‐weighted reference dose is summarized in Table 2. Quantitative comparisons of prediction errors are summarized in Figure 4 and Tables 3 and 4. Figure 4 and Table 3 present the organ‐wise MAPE, whereas Table 5 lists the corresponding MAE. The MAPE of MFMC‐DBN ranged from 1.1% to 3.9%, compared with 1.9%–6.3% for MC denoising and 3.1%–10.5% for NN. In the GTV, MFMC‐DBN achieved a MAPE of 1.98%. In the skin region, it achieved a MAPE of 3.94%, compared with 10.5% for NN. In terms of MAE, MFMC‐DBN yielded errors of 0.28 Gy (RBE) for the GTV and 0.17 Gy (RBE) for the CTV, which were lower than those of MC denoising (0.41 and 0.24 Gy (RBE), respectively) and NN (0.70 and 0.34 Gy (RBE), respectively). Relatively higher MAPE values were observed in some small or irregularly shaped OARs, such as the cochlea and eyes. This trend is partly related to the limited voxel counts in small structures and the sensitivity of MAPE to low reference dose values. Overall, the visual and quantitative results consistently indicate that MFMC‐DBN achieved the highest prediction accuracy among the three methods.

**TABLE 2:** Estimated MC statistical uncertainty (%) of the 1×10^8^‐history RBE‐weighted reference dose in target and OAR structures.

Structure	Mean ± SD	Median	95th percentile
GTV	0.79 ± 0.19	0.74	1.14
Brainstem	1.56 ± 0.07	1.47	2.31
Optic chiasm	1.27 ± 0.14	1.20	1.38
Optic nerves	1.73 ± 0.20	1.67	2.01
Cochleae	2.05 ± 0.11	2.05	2.22
Eyes	2.40 ± 0.29	2.25	3.00
Lacrimal glands	2.42 ± 0.30	2.38	2.91
Skin	2.02 ± 0.04	1.97	3.35

**TABLE 3:** Comparison of organ‐wise mean absolute percentage error for MFMC‐DBN, MC denoising, and NN. Values are reported as mean MAPE (%) with 95% confidence intervals.

	MAPE (%) with 95% Confidence Interval		
Organ	MFMC‐DBN	MC denoising	NN
GTV	1.98(1.59,2.37)	2.78 (2.31, 3.25)	4.90 (3.72, 6.07)
Skin	3.94(3.78,4.09)	6.12 (5.84, 6.40)	10.50 (9.41, 11.59)
CTV	2.03(1.74,2.32)	2.52 (2.16, 2.88)	3.80 (3.18, 4.41)
Brainstem	3.06(2.55,3.56)	4.21 (3.57, 4.86)	4.06 (3.25, 4.88)
Optic chiasm	1.10(0.62,1.58)	1.91 (1.47, 2.36)	3.19 (0.21, 6.18)
Cochlea	3.71(3.02,4.39)	6.38 (5.39, 7.37)	6.72 (5.22, 8.21)
Eyes	3.98(3.10,4.93)	5.86 (3.96, 7.76)	6.86 (5.58, 8.13)
Lacrimal glands	3.64(3.05,4.22)	5.40 (4.75, 6.05)	7.24 (4.94, 9.55)
Optic nerve	2.81(1.77,3.86)	4.11 (2.79, 5.44)	5.66 (3.46, 7.86)

**TABLE 4:** Percentage deviations of dosimetric metrics from the reference MC results for a representative test case comparing the coarse‐mesh MC and MFMC‐DBN.

Structure	Metric	Reference (Gy (RBE))	Coarse MC (%)	MFMC‐DBN (%)
GTV	D98	14.58	−86.0	−1.0
GTV	D50	33.73	−65.6	−1.6
GTV	D2	53.73	−9.5	−3.6
GTV	Mean	33.81	−49.4	−2.0
Brainstem	D98	0.47	−5.0	5.3
Brainstem	D50	1.40	10.2	3.3
Brainstem	D2	2.66	13.8	0.4
Brainstem	Mean	1.48	7.4	2.5

**TABLE 5:** Comparison of organ‐wise mean absolute error for MFMC‐DBN, MC denoising, and NN. Values are reported in Gy (RBE) with 95% confidence intervals.

	MAE (Gy (RBE)) with 95% Confidence Interval		
Organ	MFMC‐DBN	MC denoising	NN
GTV	0.28 (0.22, 0.34)	0.41 (0.31, 0.51)	0.70 (0.55, 0.82)
Skin	0.09 (0.08, 0.09)	0.10 (0.09, 0.10)	0.18 (0.17, 0.20)
CTV	0.17 (0.12, 0.22)	0.24 (0.17, 0.31)	0.34 (0.25, 0.45)
Brainstem	0.03 (0.03, 0.04)	0.05 (0.04, 0.06)	0.05 (0.04, 0.07)
Optic chiasm	0.02 (0.01, 0.03)	0.04 (0.03, 0.05)	0.07 (0.01, 0.15)
Cochlea	0.02 (0.02, 0.03)	0.04 (0.04, 0.05)	0.04 (0.03, 0.06)
Eyes	0.04 (0.03, 0.04)	0.05 (0.03, 0.06)	0.06 (0.05, 0.08)
Lacrimal glands	0.04 (0.03, 0.04)	0.06 (0.05, 0.06)	0.07 (0.04, 0.09)
Optic nerve	0.04 (0.02, 0.05)	0.06 (0.04, 0.07)	0.08 (0.03, 0.12)

**FIGURE 5:**
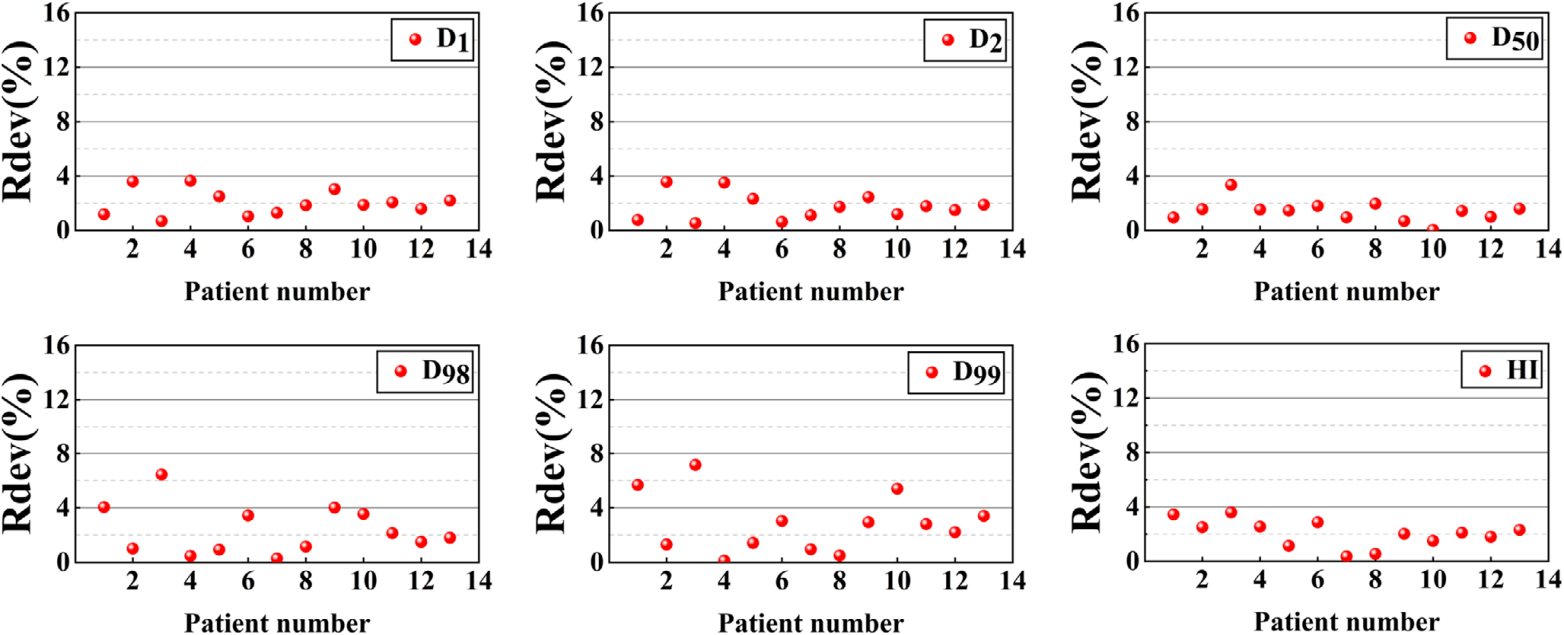
Relative deviations of GTV dosimetric parameters between MFMC‐DBN and the reference MC results for each patient, including D1, D2, D50, D98, D99, and the homogeneity index.

**FIGURE 6:**
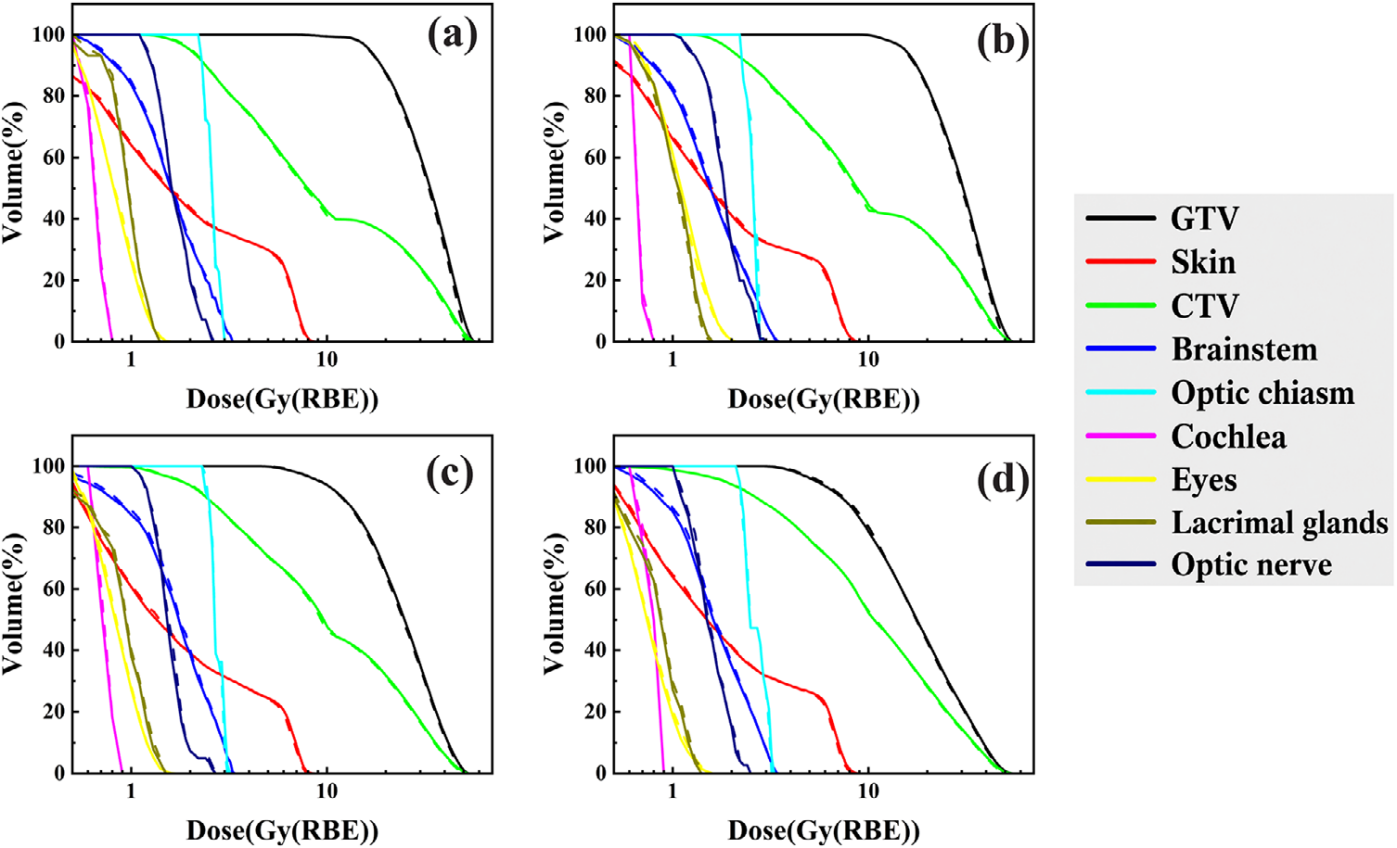
Dose‐volume histogram comparison between MFMC‐DBN and the reference MC results for four representative patients across target volumes and organs at risk. The x‐axis is displayed on a logarithmic scale.

In addition to voxel‐wise and organ‐based dose evaluations, we assessed dose‐volume histogram (DVH) agreement between MFMC‐DBN and the reference MC results. Figure 5 summarizes the patient‐wise dosimetric parameters, including D1, D2, D50, D98, D99, and HI. Figure 6 shows the DVH curves for four representative patients across different organs, indicating close agreement between the two methods. Under the 3%/3 mm criterion, gamma analysis showed passing rates of 99.8% within the patient contour, 99.3% for the GTV, and 99.7% for the CTV, indicating good spatial agreement between MFMC‐DBN and the reference MC results. The mean uncertainty was 0.79 ± 0.19% in the GTV and ranged from 1.27 ± 0.14% to 2.42 ± 0.30% across the evaluated OARs.

Slightly higher uncertainties were observed in small or low‐dose OARs, such as the cochleae, eyes, and lacrimal glands. These results indicate that residual MC statistical uncertainty was present in low‐dose OARs but remained relatively limited in the final RBE‐weighted reference dose. Because patient‐specific prescription dose information was not available in the dataset, relative deviations were used for comparison. The results show that the differences in all evaluated tumor dosimetry parameters between MFMC‐DBN and the reference MC results were small. These findings further support the dosimetric consistency of MFMC‐DBN with the MC reference.

## 4 DISCUSSION

In this study, we developed the MFMC‐DBN framework to address the time‐consuming nature of Monte Carlo (MC)‐based dose calculation in clinical BNCT. By combining low‐statistics fine‐mesh and coarse‐mesh MC results within a dual‐branch attention architecture, the proposed method enabled rapid and accurate 3D dose prediction while preserving dosimetric fidelity. The model achieved a favorable balance among prediction accuracy, computational efficiency, and interpretability under the studied conditions.

Compared with MC denoising and NN, MFMC‐DBN showed lower errors across the overall dose distribution, with improvements observed in both high‐dose and low‐dose regions. In the target regions, MFMC‐DBN achieved MAE values of 0.28 Gy (RBE) for the GTV and 0.17 Gy (RBE) for the CTV. In the skin region, the MAPE of MFMC‐DBN was 3.94%, compared with 6.12% for MC denoising and 10.5% for NN. In addition, the DVH curves generated by MFMC‐DBN closely matched those of the reference MC results. Gamma analysis further demonstrated high spatial agreement between the MFMC‐DBN predictions and the reference MC dose distributions. Under the 3%/3 mm criterion, the average passing rate within the patient contour was 99.8%. For target structures, the passing rates were 99.3% for the GTV and 99.7% for the CTV, respectively. These findings suggest that the improved performance of MFMC‐DBN is associated with the complementary integration of fine‐mesh local detail and coarse‐mesh global context through the dual‐branch design.

As shown in Table 4, the coarse‐mesh MC dose exhibited substantial deviations from the reference MC results in organ‐level dose metrics, particularly for GTV dose coverage metrics. The errors of D98, D50, and mean dose reached 86.0%, 65.6%, and 49.4%, respectively. In contrast, MFMC‐DBN reduced these errors to within 4% for all reported metrics. Similar improvements were observed for the brainstem, where MFMC‐DBN achieved closer agreement with the reference MC results than the coarse‐mesh MC dose. These findings suggest that, although the coarse‐mesh MC input provides useful global dose information, its low spatial resolution and voxel‐averaging effect limit its ability to accurately represent local dose variations and high‐gradient regions. Consequently, the coarse‐mesh MC input alone is insufficient for accurate dose evaluation. By integrating coarse‐mesh MC information with anatomical and fine‐mesh inputs, MFMC‐DBN effectively reconstructs the high‐resolution dose distribution and substantially improves dosimetric accuracy.

A major advantage of MFMC‐DBN over conventional full‐statistics MC calculation is its computational efficiency for BNCT dose prediction. In this study, PHITS simulations performed on multiple Intel Xeon Silver 4280 @ 2.10 GHz processors required approximately 70 min to complete 1 × 10^8^ particle histories using 128‐thread MPI parallelization. Under the same computational conditions, the low‐statistics MC simulation required by MFMC‐DBN used 6.5 × 10^6^ particle histories and was completed in approximately 3 min. Afterward, dose prediction on an NVIDIA RTX 4090 GPU required only a few seconds, indicating a substantial reduction in total computation time.

Although conventional NN‐based methods also require only a few seconds for inference because they do not involve MC simulation, their prediction accuracy was lower, particularly in the skin and GTV regions. The MC denoising method showed a similar overall computation time to MFMC‐DBN, but MFMC‐DBN achieved better prediction accuracy. A comparison of computational efficiency is provided in Table 6.

**TABLE 6:** Comparison of computational efficiency among full‐statistics MC, NN, MC denoising, and MFMC‐DBN, including particle histories, hardware, and computation time.

Method	Particle history	Hardware	Computation time
Full‐statistics MC	1.0×10 (8)	CPU	70 min
NN	0	GPU	1 s
MC denoising	6.5×10 (6)	CPU + GPU	3 min (MC) + 5 s (NN)
MFMC‐DBN	6.5×10 (6)	CPU + GPU	3 min (MC) + 5 s (NN)

To further assess the potential impact of volume averaging due to voxel resolution, we performed an additional PHITS dose calculation using a finer 256^3^ tally grid (voxel size 1.3 × 1.3 × 1.25 mm^3^) and compared the corresponding DVH metrics with those obtained using the original 128^3^ grid. For this comparison, we selected a representative case with a relatively steep dose gradient in order to examine a scenario in which volume‐averaging effects were expected to be more pronounced. The detailed results are summarized in Table 7, including D98, D50, and D2 for the GTV and D2 for the three OARs showing the largest relative differences. GTV DVH metrics remained highly stable, with relative differences of ‐0.46%, ‐0.04%, and ‐0.06% for D98, D50, and D2, respectively. Slightly larger deviations were observed in some small OARs, with the largest difference being 3.80% for the lacrimal gland (D2). Although these deviations remained relatively limited in this comparison, they suggest that voxel‐resolution effects are more pronounced in small structures. Therefore, when more precise dosimetric evaluation is required for clinically sensitive small organs or steep‐gradient regions, the use of a finer voxel grid may provide a more accurate local dose representation. Overall, this comparison suggests that, under the investigated conditions, voxel‐resolution effects primarily affected local dose representation in small structures, whereas their influence on the overall GTV‐level dosimetric conclusions was limited.

**TABLE 7:** Comparison of DVH metrics between 128^3^ and 256^3^ voxel grids for assessing the impact of volume‐averaging effects.

Structure	Metric	128^3^(Gy (RBE))	256^3^(Gy (RBE))	Rel. diff (%)
GTV	D98	14.58	14.51	−0.46
GTV	D50	33.72	33.70	−0.04
GTV	D2	53.73	53.69	−0.06
Lacrimal glands	D2	1.05	1.09	3.80
Cochlea	D2	0.64	0.66	3.26
Skin	D2	7.84	8.04	2.50

This study demonstrates the feasibility of the MFMC‐DBN framework for 3D BNCT dose calculation under the specific irradiation setting investigated here. In particular, the model was developed and evaluated using fixed neutron incident angles and source‐to‐skin distances. Therefore, the current results should be interpreted within this restricted geometric setting rather than as evidence of full generalizability to arbitrary treatment configurations. Further validation using more diverse irradiation geometries, including different beam angles, isocenter positions, and source‐to‐skin distances, will be necessary before the proposed framework can be extended to more general clinical treatment‐planning scenarios.

Another limitation is related to the spatial resolution of the dose representation. The selected fine‐mesh voxel size of 2.6 × 2.6 × 2.5 mm^3^ is larger than the voxel sizes commonly used in clinical treatment planning and may introduce volume‐averaging effects in small structures and steep dose‐gradient regions. If results closer to clinical practice are required, a finer mesh division would still be necessary. This limitation is expected to be more pronounced in the coarse‐mesh representation. However, in the proposed framework, the coarse‐mesh dose is not used as the final dose output, but only as auxiliary global guidance for the network. Local dose details are primarily provided by the fine‐mesh input and preserved through the main branch of the model. Therefore, although the coarse mesh may not fully represent the fine spatial characteristics of very small OARs and may partly contribute to reduced prediction accuracy in these structures, it is not intended to determine their final local dose representation. Moreover, because the reference dose distributions used for network training were generated on the same 2.6 × 2.6 × 2.5 mm^3^ voxel grid, the final prediction target was defined at this spatial resolution. Thus, volume averaging should be regarded mainly as a limitation inherited from the voxel resolution of the reference dose representation, rather than as an additional distortion introduced by the neural‐network framework itself. In addition, the overall improvement of MFMC‐DBN compared with the MC denoising method suggests that, under the investigated conditions, incorporating coarse‐mesh information did not noticeably compromise the overall dose prediction performance and instead provided useful low‐fidelity guidance for the network.

Prediction accuracy in low‐dose regions remained lower than that in high‐dose target regions, especially for some small OARs receiving very low doses. In these cases, relatively high MAPE values may arise even when the corresponding absolute dose differences are small, because the denominator in the relative error calculation is also small. In addition, the use of standard MSE as the training loss may have contributed to this tendency. Since MSE places greater emphasis on voxels with larger absolute dose errors, the training process may preferentially optimize high‐dose regions, whereas low‐dose regions contribute less to the overall loss. This property may partly explain the relatively higher MAPE values observed in distal or low‐dose OARs. Furthermore, the estimated uncertainty analysis showed that the 1 × 10^8^‐history RBE‐weighted reference dose had slightly higher residual MC statistical uncertainty in small or low‐dose OARs than in the GTV. Therefore, the relatively higher MAPE values in these structures may reflect not only model prediction error but also residual statistical uncertainty in the reference MC data. Nevertheless, the estimated uncertainty of the final RBE‐weighted reference dose remained limited, suggesting that the observed prediction errors were unlikely to be dominated solely by MC noise in the reference data. The high gamma passing rates further support that the proposed framework preserves both overall dosimetric accuracy and local spatial consistency. Although these low‐dose discrepancies may be less influential than target‐region errors in many treatment‐planning scenarios, they remain an important limitation of the current framework.

For broader clinical applicability, a larger and more diverse training dataset will be required to include dose distributions generated under different beam angles, source‐to‐skin distances, irradiation conditions, and anatomical sites. Extending the proposed framework to multiple beam angles and source‐to‐skin distances is expected to be feasible because the network learns the mapping between low‐statistics and high‐accuracy MC dose distributions rather than a specific irradiation geometry. The primary challenge is expected to be the generation of sufficiently diverse training data covering a broader range of treatment conditions. Additional sensitivity analyses will also be needed to quantify the robustness of the model to such geometric variations. The present study focused on 112 glioblastoma patients with relatively fixed head‐and‐neck geometry because of dataset availability. Therefore, the current results should be interpreted within this restricted setting rather than as evidence of full generalizability to arbitrary treatment configurations. To extend the proposed framework to varying irradiation geometries, future models should incorporate explicit geometry‐aware information. For example, beam angle, beam direction, source‐to‐skin distance, source position, or isocenter location could be encoded as additional scalar inputs or spatial input channels. Spatial geometry maps, such as a beam‐direction map, source‐position map, or distance‐to‐beam‐axis map, may allow the network to learn the relationship among patient anatomy, irradiation geometry, and the resulting dose distribution. Transfer learning or configuration‐specific fine‐tuning may also be useful when extending the model to new beam settings or treatment sites.

In future work, we will investigate multi‐directional dose prediction based on multi‐objective optimization algorithms^23^ and integrate the proposed framework with multi‐beam optimization methods to support faster and more clinically practical BNCT treatment planning. In addition, multi‐head network architectures will be investigated to reduce model size and inference overhead while enabling simultaneous prediction of the four BNCT dose components.

## 5 CONCLUSIONS

In this study, we developed and validated MFMC‐DBN, a deep learning framework designed to address the time‐consuming nature of dose calculation in clinical BNCT. By using dual‐branch attention architecture to fuse complementary information from low‐statistics coarse‐mesh and fine‐mesh MC simulations, the model achieved both high computational efficiency and accurate dose prediction. Evaluation on a clinical dataset of glioblastoma patients showed that MFMC‐DBN outperformed conventional NN and MC denoising methods. The model achieved a MAPE of 1.98% in the GTV while maintaining close agreement with the reference MC results. Gamma analysis further confirmed good spatial agreement, with passing rates of 99.8% within the patient contour and 99.3% in the GTV under the 3%/3 mm criterion. In addition, the total dose‐calculation time was reduced from approximately 70 min for full MC simulation to about 3 min for the low‐statistics MC step, followed by only a few seconds for neural‐network inference.

These results support the feasibility of MFMC‐DBN for accelerated BNCT dose calculation under the studied irradiation setting. The proposed framework shows promise for future integration into BNCT treatment‐planning workflows after broader validation under more diverse beam geometries and clinical conditions. Future work will focus on extending the model to varying beam angles, broader anatomical sites, and more clinically diverse scenarios, as well as on improving its compatibility with treatment‐planning‐system implementation. In addition, future studies will evaluate finer‐resolution dose‐scoring strategies and voxel‐size sensitivity to further assess and reduce the impact of volume averaging, particularly in small OARs and steep dose‐gradient regions.

## CONFLICT OF INTEREST STATEMENT

The authors declare no conflicts of interest.

## Data Availability

The data that support the findings of this study are available from the corresponding author upon reasonable request.
